# Temporal stability of the rumen microbiota in beef cattle, and response to diet and supplements

**DOI:** 10.1186/s42523-019-0018-y

**Published:** 2019-11-19

**Authors:** Timothy J. Snelling, Marc D. Auffret, Carol-Anne Duthie, Robert D. Stewart, Mick Watson, Richard J. Dewhurst, Rainer Roehe, Alan W. Walker

**Affiliations:** 1 Rowett Institute, University of Aberdeen, Aberdeen, AB25 2ZD UK; 2 SRUC, Kings Buildings, Edinburgh, EH9 3JG UK; 3 The Roslin Institute and R(D)SVS, University of Edinburgh, Edinburgh, EH25 9RG UK

**Keywords:** Rumen, Microbiota, Concentrate diet, Forage diet, Nitrate supplementation, Oil supplementation, Temporal stability

## Abstract

**Background:**

Dietary intake is known to be a driver of microbial community dynamics in ruminants. Beef cattle go through a finishing phase that typically includes very high concentrate ratios in their feed, with consequent effects on rumen metabolism including methane production. This longitudinal study was designed to measure dynamics of the rumen microbial community in response to the introduction of high concentrate diets fed to beef cattle during the finishing period.

A cohort of 50 beef steers were fed either of two basal diet formulations consisting of approximately 10:90 or 50:50 forage:concentrate ratios respectively. Nitrate and oil rich supplements were also added either individually or in combination. Digesta samples were taken at time points over ~ 200 days during the finishing period of the cattle to measure the adaptation to the basal diet and long-term stability of the rumen microbiota.

**Results:**

16S rRNA gene amplicon libraries were prepared from 313 rumen digesta samples and analysed at a depth of 20,000 sequences per library. Bray Curtis dissimilarity with analysis of molecular variance (AMOVA) revealed highly significant (*p* < 0.001) differences in microbiota composition between cattle fed different basal diets, largely driven by reduction of fibre degrading microbial groups and increased relative abundance of an unclassified *Gammaproteobacteria* OTU in the high concentrate fed animals. Conversely, the forage-based diet was significantly associated with methanogenic archaea. Within basal diet groups, addition of the nitrate and combined supplements had lesser, although still significant, impacts on microbiota dissimilarity compared to pre-treatment time points and controls. Measurements of the response and stability of the microbial community over the time course of the experiment showed continuing adaptation up to 25 days in the high concentrate groups. After this time point, however, no significant variability was detected.

**Conclusions:**

High concentrate diets that are typically fed to finishing beef cattle can have a significant effect on the microbial community in the rumen. Inferred metabolic activity of the different microbial communities associated with each of the respective basal diets explained differences in methane and short chain fatty acid production between cattle. Longitudinal sampling revealed that once adapted to a change in diet, the rumen microbial community remains in a relatively stable alternate state.

## Background

As a result of increasing demand for meat and milk, particularly in developing countries, ruminant livestock production is becoming one of the fastest growing agricultural sectors [1]. This trend has led to concerns regarding environmental impact, where livestock farming currently accounts for 44% of the total anthropogenic sources of the greenhouse gas methane (CH_4_) [2]. Ruminant recovery of energy from the diet, as well as the production of CH_4_ and N_2_O, is due to the activity of the rumen microbial community. Therefore, manipulation of the microbiota has the potential to improve the efficiency of animal production, and mitigate greenhouse gas emissions [3]. A practical approach to achieve this is by management of the dietary intake.

The components making up a typical basal diet fed to beef cattle can be categorised into two major feed types. Plant fibre, including straw, hay, and grass or cereal crop silage are classified as forage. A variety of feeds, typically pelleted, and composed of nutrient-rich grains, starch, sugars or protein are classified as concentrates. The different ratios of the two feed types can influence the composition of the rumen microbial community both as a response to the different carbohydrate sources in the diet [4] and as a result of the changes in interactions between microbial groups [5]. In turn this alters the production rates of microbial metabolic products including short chain fatty acids (SCFA) and methane [6, 7].

Supplements are added to the diet to improve performance or reduce methane production according to key principles. Firstly, they can directly influence growth of key members of the microbial community, either promoting growth of beneficial microbes or inhibiting growth of detrimental microbes. For example, addition of oils can have a defaunating effect, and at high doses can reduce or eliminate ciliate protozoa in the rumen [8], and vegetable oils and fish oils at doses as low as 1–2% have been found to directly affect the growth of key bacterial species [9]. Nitrate is added as a theoretical ‘sink’ for hydrogen (H_2_) where it is reduced, typically by *Selenomonas* spp., to nitrite and ultimately to ammonia [10]. Reduction of ruminal hydrogen in theory limits substrate availability for growth of the methanogenic archaea [11]. Secondly, the supplement can have an effect on metabolite production by inhibiting the activity of key enzymes involved in certain metabolism pathways. Examples are the nitrooxy compounds that inhibit catalysis of the final step of the methanogenesis pathway [12].

The effect of nitrate and oil/fatty acid supplements on ruminal methane production has not always been reflected by associated changes in the microbial community [13–16]. The outcomes of these studies are highly dependent on the dose and the chemical composition of the nitrate salt [10], as well as the choice of methodology, sensitivity of the measurements, the power of the statistical analysis and factoring of variability of responses between individual experimental animals. In studies involving larger cohorts of experimental animals, combined with the appropriate discriminant analysis of metagenome datasets, it has been possible to identify rumen microbe functional biomarkers and inferred taxonomic groups for methane emissions in response to both basal diets and supplements [17].

Longitudinal experiments have been carried out previously to monitor the development of rumen microbiota during early life [18, 19], to measure the temporal dynamics of the microbiota during colonisation and breakdown of dietary fibre [20–22] and the diurnal variability [23]. However, there is a lack of knowledge on the long-term stability and repeatability of measurements of the microbial community during the finishing phase of mature livestock animals.

The aim of the current study was to characterise the rumen microbial community of beef cattle in response to two basal diets comprising different forage:concentrate ratios with addition of high oil and nitrate supplements over the course of the finishing stage of production. Longitudinal sampling enabled the measurement of the temporal dynamics and stability of the microbial community over this period. The most significant discriminant groups of microorganisms responsible for driving changes as a response to diet over time were identified.

## Results

16S rRNA gene sequencing of rumen digesta samples was used to assess potential links between basal diet, rumen microbiota composition, and host animal measures such as methane emission and feed efficiency. In total, 313 16S rRNA gene amplicon libraries were sequenced using rumen samples collected during two feed trials carried out over consecutive years (2013 and 2014). 50 finishing beef steers (32 in 2013 and 18 in 2014) were sampled periodically at time points covering the seven-month finishing period when the animals are fed to gain weight and optimise meat and fat composition prior to slaughter. Sampling time points were evenly distributed (approximately one month apart) and covered critical time points listed in Table 1.

**Table 1:** Sampling timetable for (a) NutriBeef 2013 and (b) NutriBeef 2014 diet and supplement trials

Time point	(day)	Date	Description
(a)			
TP0	0	30th May 2013	Pre-treatment
TP1	25	24th June 2013	Adaptation
TP2	42	11th July 2013	Start test
TP3	74	12th August 2013	Mid test
TP4	102	9th September 2013	End test
TP5	111–189	18th September – 5th December 2013	Chamber
TP6	126–195	3rd October – 11th December 2013	Slaughter
(b)			
TP0	0	14th April 2014	Pre-treatment
TP1	14	28th April 2014	Adaptation
TP2	31	15th May 2014	Start test
TP4	98	21st July 2014	End test
TP5	114–199	6th August – 30th October 2014	Chamber
TP6	141–203	2nd September – 3rd November 2014	Slaughter

TP0: Pre-treatment: After transition to the Forage and Concentrate diets but before introduction of Nitrate and Lipid treatments

TP1: Adaptation: Seven days after introduction to Nitrate and Lipid treatments when cattle were being offered 25% of the maximum dose of Nitrate

TP2: Start test: At the start of the 56 day performance test period

TP3: Mid test: At the mid-point of the 56 day performance test period (2013 samples only)

TP4: End test: At the end of the 56 day performance test period

TP5: Chamber: After completion of the 56 day performance test period, when each animal left the respiration chambers over a period of 13 weeks (six animals/week)

TP6: Slaughter: At the abattoir, when cattle were slaughtered in four batches

**Fig. 1:**
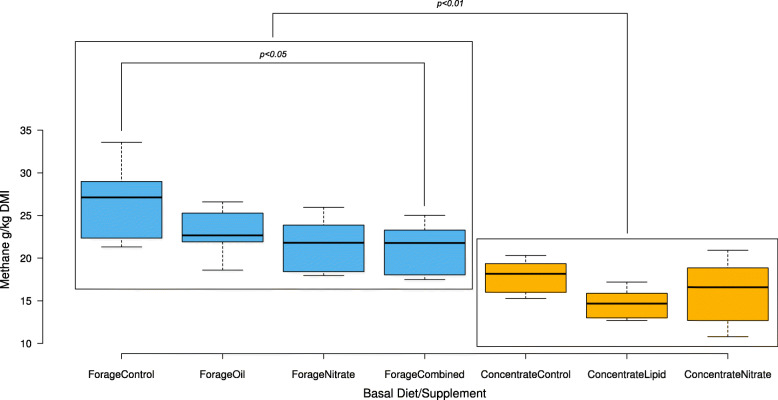
Effect of basal diet and supplement on methane emissions measured using respiration chambers. Methane values are expressed as g per kg dry matter intake (DMI). Methane emissions were significantly lower in concentrate fed compared to forage fed cattle (*p* < 0.01). Cattle fed forage diets with combined nitrate supplementation showed significantly lower methane emissions compared to forage controls (*p* < 0.05). Data collected from animals fed a forage-based diet are indicated in blue, and those from animals on a concentrate diet are shown in orange

Results previously reported in Troy et al., (2015) and Duthie et al., (2018) [7, 24] from the same animal cohort, found basal diet and, to a lesser extent, nitrate and oil supplements had a significant effect on average methane production across all the animals. The high concentrate diet was associated with significantly lower (*p* < 0.001) CH_4_ emissions g per kg Dry Matter Intake (DMI) (Fig. 1), as well as lower molar proportions of acetate (*P* < 0.001) and butyrate (*P* < 0.01) and higher molar proportions of propionate (*P* < 0.001) and valerate (*P* < 0.05) [7]. Within basal diets, only the combined nitrate and oil supplementation significantly reduced methane production compared to control in the forage fed cattle (Fig. 1). Average residual feed intake (RFI) was lower, (i.e. higher efficiency) in the high concentrate fed animals. However, this was not considered statistically significant.

After quality control and subsampling, 16S rRNA gene sequencing resulted in a total of 6.26 million sequences (randomly subsampled to 20,000 per library) for further analysis, providing > 97% species coverage (Good’s Coverage Index) for each sample. A total of 12,361 OTUs were identified, with over 99% of the sequences contained in the top 2000 OTUs. SILVA 128 SEED reference taxonomy assigned the majority of sequences (39%) to the *Bacteroidetes*, 30% to *Firmicutes*, 16% to *Gammaproteobacteria*, 6% to the *Euryarchaeota*, 2% *Actinobacteria*, 2% *Spirochaetes* 1% *Verrucomicrobia* and 1% *Fibrobacteres*, with 3% unclassified phyla (Additional file 1: OTU Table). Mean proportional compositions at the family level for both forage and concentrate-fed animals are shown in Additional file 2: Figure S1.

**Fig. 2:**
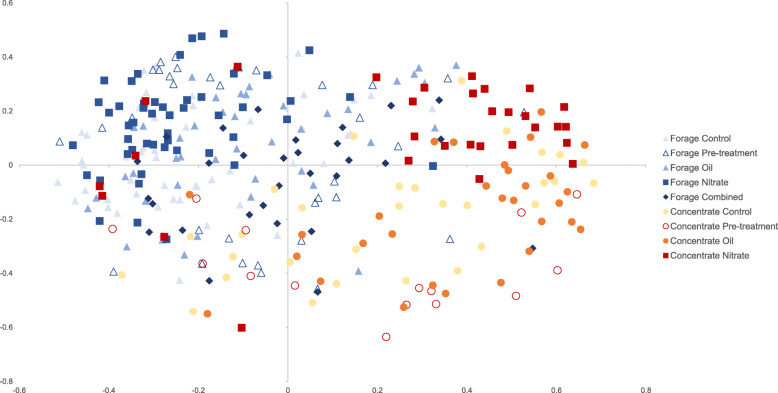
NMDS plot (Stress value 0.21) based on Bray Curtis dissimilarity matrix showing significant clustering of samples by basal diet (*p* < 0.001, AMOVA calculated using mothur software)

Average microbial diversity as assessed by calculating the Shannon index for each sample, was significantly lower (*p* < 0.001) in the cattle fed a high concentrate basal diet (Additional File 2: Figure S2). Furthermore, analysis of molecular variance (AMOVA) applied to the distance matrix used for the non-metric multidimensional scaling plot (NMDS) revealed highly significant clustering of microbial communities by basal diet (*p* < 0.001) (Fig. 2). Within each of the two basal diet groups, the microbial communities clustered significantly (*p* < 0.001) following nitrate supplementation compared to all other treatment and control groups. In the forage basal diet groups, combined oil/nitrate supplement samples (2013) clustered separately from the control group only (*p* < 0.001). In the concentrate fed animals, the oil supplement samples separated from the pre-treatment samples (*p* < 0.001). There was no significant microbial community dissimilarity between control animals and pre-treatment groups (Additional file 2: Table S3).

**Fig. 3:**
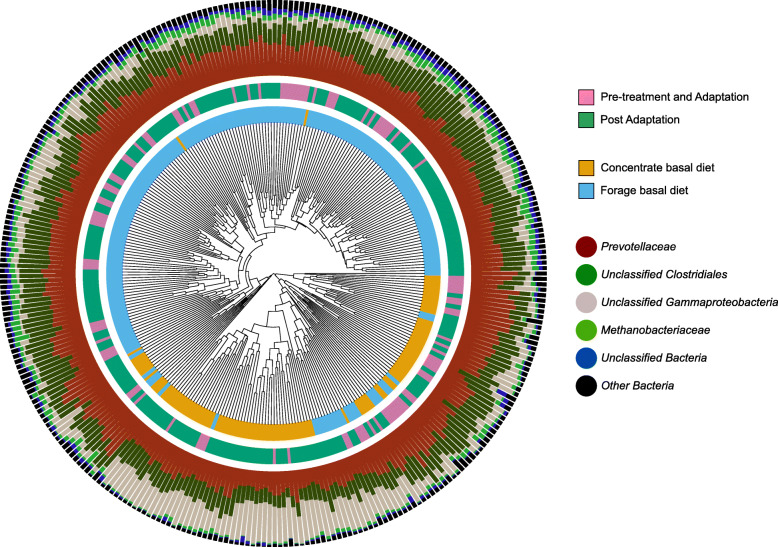
Dendrogram based on Bray Curtis dissimilarity matrix showing strong clustering between animals fed concentrate and forage basal diets. Clustering was largely driven by increased relative abundance of Unclassified *Gammaproteobacteria* following pre-treatment/adaptation time points. Inner Ring: Basal Diet. Middle Ring: Timepoints, including Pre-treatment and Adaptation (TP0 – TP1) and Post Adaptation (TP3 to Slaughter). Outer Ring: Taxon proportional abundance (Family)

Supporting the AMOVA results, significant clustering of the microbial communities of forage and concentrate fed animals were also observed when using parsimony analysis of the microbial community dendrogram (Fig. 3). This clustering was largely driven by an increase in relative abundance of a single species-level operational taxonomic unit (OTU) (OTU00001, assigned to *Gammaproteobacteria*) in concentrate fed animals, which was also particularly associated with the later time points following the pre-treatment and adaptation periods.

Analysis of similarities (ANOSIM) confirmed the AMOVA results with highest R values associated with comparisons between animals fed different basal diets (*p* < 0.001). Nitrate (2013 and 2014) and oil (2013 and 2014) supplementation appeared to have relatively lower R values, but nonetheless significant, effects (*p* < 0.001). The only non-significant treatment was the oil in conjunction with the forage diet when compared to pre-treatment time points. No significant effects or interactions were found as a result of breed or trial year within treatments.

Linear discriminant analysis (LDA) using LEfSe software [25] identified highly significant OTU biomarkers associated with basal diet groups. With minimum linear discriminant analysis (LDA) effect size set at threshold > 4.0, species level OTUs were assigned to the lowest taxonomic level (using SILVA 128 classification at 100% confidence), with *Gammaproteobacteria* (class), *Prevotella* (genus) and *Phascolarctobacterium* (genus) significantly associated with high concentrate basal diets. OTUs assigned to *Methanobrevibacter* (genus), *Ruminococcacaeae* (family), *Proteobacteria* (phylum) and *Lachnospiraceae* (family) were significantly associated with forage basal diets (Table 2).

**Table 2:** OTU level taxonomic biomarkers for (a) concentrate basal diets and (b) forage basal diets (Linear discriminant analysis effect size > 4.0)

(a)		
Concentrate	% Seq	SILVA 128 Taxonomy (Mixed Rank)
OTU00001	10.5	Unclassified *Gammaproteobacteria* (Class)
OTU00004	2.6	*Prevotella* (Genus)
OTU00017	1.0	*Phascolarctobacterium* (Genus)
OTU00019	0.9	*Prevotella* (Genus)
(b)		
Forage	% Seq	SILVA 128 Taxonomy (Mixed Rank)
OTU00003	2.9	*Methanobrevibacter* (Genus)
OTU00006	2.3	*Ruminococcaceae* (Family)
OTU00005	2.4	*Proteobacteria* (Phylum)
OTU00009	1.9	*Lachnospiraceae* (Family)

Taxonomic classification to the lowest identified rank assigned using SILVA SEED 128 reference database with bootstrap values of 100. % Seq: Proportion of sequence counts from total dataset representing each OTU

Taxonomic biomarkers were also found to be associated with the animals in the upper quartile of feed efficiency RFI at less stringent effect sizes (LDA > 2.0). These OTUs were low proportional abundance (typically 0.1–0.3% of the total microbial population), however, and were identified as species from the *Prevotellaceae*, *Rikenellaceae* and *Acidaminococcaceae* families.

**Fig. 4:**
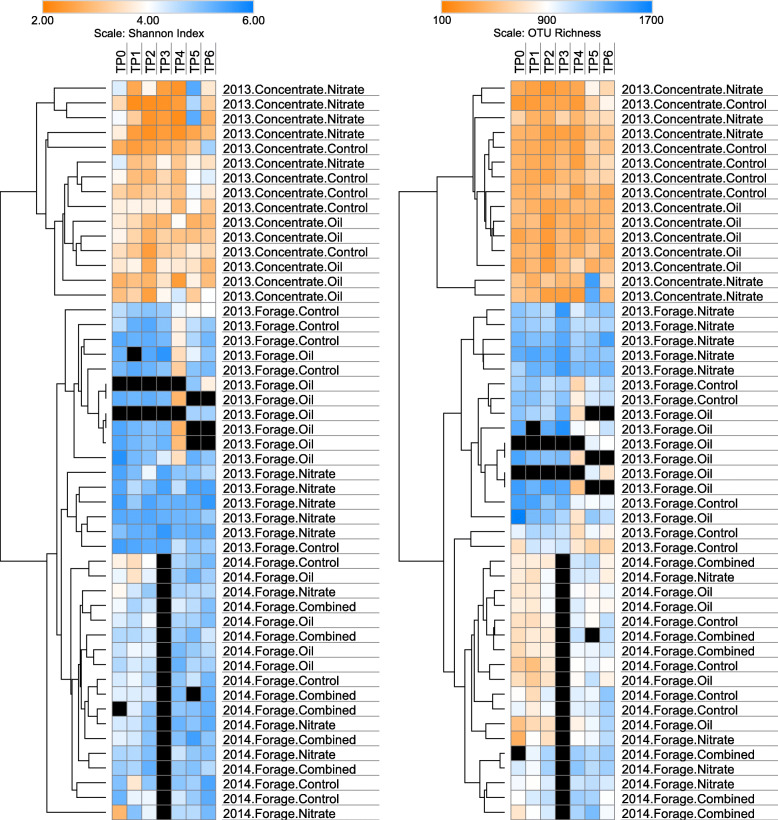
Temporal stability of microbial community alpha diversity based on (a) Shannon diversity index and (b) OTU richness measurements. Time Points: TP0 – Pre-treatment, TP1 Adaptation, TP2 – Performance Test Start, TP3 – Performance Test Mid, TP4 – Performance Test End, TP5 – Methane Chamber, TP6 – Slaughter. Clustering: Euclidean distance of average values. Black cells: Data not available

Next, we carried out a longitudinal analysis, in order to assess the temporal stability of the rumen microbiota. Microbial community alpha diversity appeared to be relatively stable across sampling times during the ~ 200 days from Pre-treatment (TP0) to Slaughter (TP6), with the concentrate fed animals again showing consistently lower rumen microbiota diversity than those on the forage diet (Additional file 2: Figure S3). Clustering by Euclidean distance of average values of observed species richness (SOBS) and Shannon diversity index (H′) across the total time course separated samples by basal diet, with lower microbial community diversity in the high concentrate fed animals compared to the forage group. Within the forage fed animals, average alpha diversity was strongly influenced by trial year, with most animals from 2013 containing significantly lower average species richness and average microbial diversity (Shannon index) from those in the 2014 trial (*p* < 0.001) (Fig. 4, Additional file 2: Figure S4). However, discriminant analysis (LDA effect size > 4.0) did not reveal any significant OTUs between the 2013 and 2014 forage fed groups.)

**Fig. 5:**
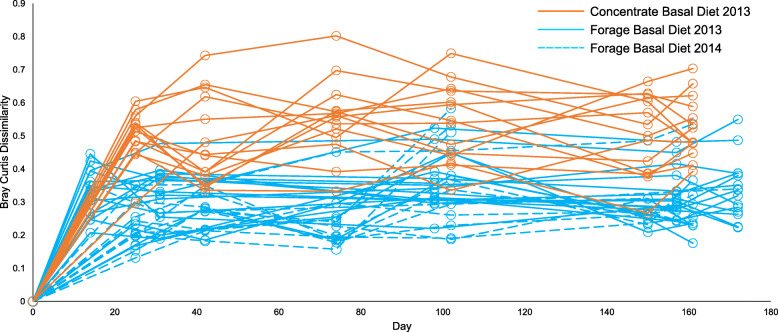
Temporal stability of microbial community beta diversity (Bray Curtis dissimilarity) over time (Days). Comparison of basal diets (all supplements and control treatments). Significant microbial community dissimilarity (*ANOVA *p* < 0.001) was only observed between time point TP0 (Day 0) and TP1 (Day 25: 2013 Animal trial)

Response and adaptation of the microbial community to the basal diets/supplements was measured using the Bray Curtis metric, which measured community dissimilarity relative from the pre-treatment time point as a baseline. Repeated measures ANOVA on these data showed significant community dissimilarity only occurring between Pre-treatment (TP0) and Adaptation (TP1) time points in high concentrate fed cattle. Forage fed cattle from both 2013 and 2014 animal trials did not show significant variability in microbial community composition regardless of additional supplement (Fig. 5). Pairwise Spearman correlations of Bray Curtis values between these time points were all highly significant (*p* < 0.001) (Additional file 2: Figure S6). Furthermore, the higher average dissimilarity values associated with concentrate diets compared to forage diets in both trial years indicated that the initial addition of concentrates to the diet preceded sweeping changes in microbiota composition. There was no further subsequent significant change in dissimilarity over time points TP1-TP6, however, indicating that the rumen microbiota remained relatively stable after adaptation to a given diet.

**Fig. 6:**
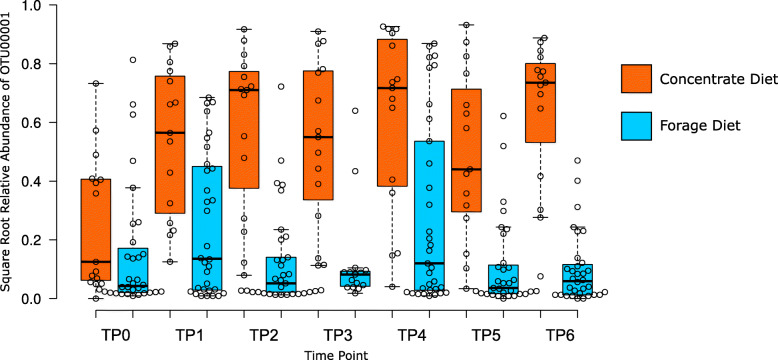
Temporal response and stability of OTU00001 (Unclassified Gammaproteobacteria*) in rumen samples of animals fed a high concentrate basal diet. x-axis: Time Points: TP0 – Pre-treatment, TP1 Adaptation, TP2 – Performance Test Start, TP3 – Performance Test Mid, TP4 – Performance Test End, TP5 – Methane Chamber, TP6 – Slaughter. y axis: Square root transformed relative abundance of sequence counts. *SILVA 128 Taxonomic classification

Of the OTU biomarkers associated with basal diets (LDA effect size > 4.0), only OTU00001 (derived from an uncultured *Gammaproteobacteria* lineage) showed significant temporal response to the introduction of the high concentrate diet. The relative abundance of this OTU increased upon addition of concentrates to the diet, and its dominance was maintained throughout the remainder of the experimental period while concentrates were being continually fed to the animals (Fig. 6).

## Discussion

The effects on the rumen microbial community of basal diet, and addition of nitrate and high-oil supplements, was determined with 50 experimental animals consisting of mature beef cattle during the seven-month finishing period.

High concentrate diets were significantly associated with lower methane emissions (g/kg DMI), decrease in acetate and an increase in propionate production. The change in metabolite production was in turn associated with better feed efficiency/lower residual feed intake (RFI). This supported the theory that energy stored in metabolites such as methane may represent a loss to the animal.

The Shannon diversity and species richness of the microbial communities was significantly lower in the concentrate fed compared to the forage fed groups. The effect of microbial community diversity on feed efficiency was previously investigated by Kruger Ben Shabat et al., (2016) [26], who reported that decreased microbial diversity was associated with a decrease in energy requirements needed for the production of non-relevant metabolites.

The cohort including the forage fed animals were split over two trial years (2013 and 2014), and some apparent differences in microbial diversity were revealed between these two groups, driven in part by a difference in the OTU richness. Analysis of the temporal stability of the microbial community accounted for the difference in baseline diversity as part of the statistical model and revealed a highly significant response over time of the rumen microbial community to the introduction of high concentrate diets compared to the forage fed groups from both trials. This response was followed by relative stability and illustrated in real time the perturbation, and re-stabilisation of the rumen microbial community after a given period of adaptation. Moreover, discriminant analysis provided compelling evidence of the most important microbial groups driving these changes.

Four sequence classification groups accounted for 90% of the total community: The *Prevotellacaceae* family (39%), sequences derived from uncultured organisms that could only be confidently assigned to the *Clostridiales* order (29%) and *Gammaproteobacteria* class (16%), and the archaeal family *Methanobacteriaceae* (6%). Changes in the relative abundance of these groups likely reflected their substrate preferences and/or tolerance of the environmental conditions [27] as a result of the different diets provided to the cattle.

Interestingly, the proportional abundance of the family *Prevotellaceae* relative to the total community did not change in the high concentrate animals. In contrast, the *Methanobacteriaceae* decreased in favour of increased relative abundance of unclassified *Gammaproteobacteria* (Additional file 2: Figure S1). This suggests that the growth of the unclassified *Clostridiales* was less competitive in a low fibre environment, whereas the *Prevotellaceae* have a greater degree of adaptability to gut conditions [28].

Hydrogenotrophic *Methanobrevibacter* spp. were associated with the forage fed animals, and this was reflected in significantly higher methane production (g CH_4_ /kg DMI) in this group. Reduction of methane emissions relative to dry matter intake (g/kg DMI) is a widely reported effect of increasing percentage of concentrate in the diet formulation, particularly at levels exceeding 80–90% concentrate:forage ratios [29]. This is typically associated with a shift from acetate toward propionate production, as was the case in the present study and previously reported in Troy et al., (2015) [7] and Duthie et al., (2018) [24].

A single *Proteobacteria* OTU (OTU00005) was associated with forage diets. SILVA 128 taxonomy was unable to classify this uncultivated species in more detail. However, an NCBI BLASTn type search and the RDP classifier most closely aligned the representative sequence to a member of the *Pasteurellaceae*. This family includes rumen isolates *Basfia succiniciproducens* [30] and *Actinobacillus succinogenes* [31], both characterised as succinate producers with the ability to reduce nitrate. It is possible that the uncharacterised OTUs detected here might have similar functionality. In support of this, LEfSe analysis within diet groups confirmed its association with the nitrate treatment.

The single most abundant OTU, comprising over 10% of total sequence counts, was significantly associated with the high concentrate basal diet. The representative sequence was assigned to the *Gammaproteobacteria* class using SILVA 128 reference taxonomy, but was not identified as any known cultured isolate. Using the Greengenes reference database, the sequence was mapped to *Succinivibrionaceae* (71% bootstrap support), while the RDP classifier and an NCBI BLAST type search to mapped it to the genus *Frischella* (65% bootstrap support and 89% sequence identity respectively) [32]. Phylogenetic analysis (Additional file 2: Figure S5) placed it in a group containing the novel *Orbales* order isolated from the gut of insects [33]. Examples of rumen *Gammaproteobacteria* isolates *Actinobacillus succinogenes*, *Basfia succiniciproducens* and *Mannheimia succiniciproducens* [30, 31, 34] are all known for their ability to produce succinate.

Isolates of the succinate-producing *Succinivibrionaceae* family of microbes have also been characterised from the foregut of the Tammar wallaby, a herbivore known for its unusually low methane emissions [35]. In a previous metagenomic analysis, *Succinivibrionaceae* were associated with low methane emitting phenotypes in beef cattle [36]. In the present study, detailed taxonomic classification of the *Proteobacteria* OTU sequences beyond class level was limited by the lack of available references. However, an NCBI BLASTn search of the representative sequences against the total current nucleotide database found many hundreds of highly similar sequences previously recovered from ruminants but not identified. This indicates that this species is likely to be an important and widespread rumen microbiota constituent, and highlights the importance of continuing efforts to culture, isolate and better characterise the rumen microbiota [37]. Cumulatively, the sequence-based data suggest that there are important groups of unclassified *Proteobacteria*, possibly containing novel taxa that may have a significant role in rumen methane emissions.

Following discriminant analysis, two OTU biomarkers assigned to *Prevotella* spp. were strongly associated with the high concentrate diet group. Isolates from this genus are known to produce propionate via the succinate pathway [38], although with low pH conditions some species of *Prevotella* can be associated with accumulation of succinate [39]. In response to these conditions, a prominent succinate consumer would be expected to thrive. In this study, an uncultured *Firmicutes* organism strongly associated with the concentrate diets was identified as *Phascolarctobacterium* (SILVA) or *Succiniclasticum* (Greengenes/RDP/BLASTn). The type species of this genus, *Succiniclasticum ruminis*, is known to produce propionate from succinate as the sole mechanism of energy production [40]. Short chain fatty acid analysis, carried out previously, confirmed significantly lower acetate to propionate ratio in concentrate fed animals [7, 24].

No archaea were associated with high concentrate diets above our arbitrarily high LEfSe-based linear discriminant analysis threshold. However, at lower LDA effect size settings, OTUs classified as *Methanobrevibacter boviskoreani,* a methanogenic archaeal species recently isolated from the rumen of Korean cattle [41] and *Methanomassiliicoccaceae*, a methylotrophic methanogen group previously associated with low methane emissions in the rumen [42], were both significantly associated with high concentrate diets/low methane emissions in the cattle.

Previous comparable analyses of the human gut microbiota have established that, in the absence of major perturbations, the most abundant groups of the microbial community remain in a largely stable state [43, 44]. In contrast, previous longitudinal studies of the rumen microbiota indicate that it can vary significantly over long term seasonal time scales, probably as a result of the changes in grazing quality throughout the year [45]. In the short term, the diurnal variability of the rumen microbial community can overpower both individual and diet effects. The latter effect is typically seen when high concentrate containing rations are provided to the animal once per day [23]. The principle of short-term variability as a result of dietary effect, followed by long term stability was broadly supported in this study. Following the period of adaptation and change, an alternative stable microbial community state was established for the duration of the feed trial. This type of response and single alternative state is one of the models used to describe the variability of community types in response to a change in environment. Other models such as multi-stability or selection of local communities described in the human gut have not been described in the rumen [46].

Significant changes in the composition of the rumen microbiota can arise as a result of changes in diet promoting increase of the taxa that can best utilise these substrates for metabolism. At the extreme levels of concentrate to forage (90:10) typically provided as a beef cattle finishing diet, reduction of key fibre degraders would be expected [47]. In agreement with this expectation, in the current study LEfSe-based linear discriminant analysis of taxa revealed key fibre-degrading *Clostridiales* species were most negatively impacted by the dietary change, whereas more generalist taxonomic groups such as *Prevotella* spp. appeared to be relatively unaffected.

Another mechanism by which dietary changes might change the rumen microbiota is a niche modification effect, whereby the bacteria themselves alter their environment, affecting growth of functionally associated groups [45]. A possible niche modification following dietary change is indicated by the significant increase in proportional abundance of *Succiniclasticum*, possibly as a result of accumulation of its preferred growth substrate succinate, and a significant decrease in *Methanobrevibacter*, likely as a result of reduced availability of hydrogen. The dramatic increase of concentrates favoured a single unclassified species (OTU) of *Gammaproteobacteria.* Linear discriminant analysis identified this OTU as the only taxon significantly associated with the change of the microbiota between TP0 and TP1. As this organism is currently uncultured, it is unknown whether its proportional increase was driven directly by diet effects, or indirectly via niche modification.

## Conclusions

Our results build on existing studies emphasizing the importance of diet, and in particular the ratio of concentrate to forage, in driving the composition of the rumen microbiota [4–6, 17, 23]. Changes in the ruminal microbiota composition following addition of high concentrate diets and supplements explained many of the phenotypic changes previously reported in the rumen, including methane emissions and SCFA production [24]. Microbial community changes were largely driven by a small number of highly proportionally abundant OTUs, with one identified as an uncultured member of the *Gammaproteobacteria* of particular significance. As assessed by longitudinal sampling, the change in relative abundance of this and other corresponding taxa was observed during the initial response and the adaptation period. This was followed by a period of relative stability, in respective alternative states corresponding to either the forage or high concentrate diets. This result is reassuring for cross-sectional studies as our results suggest that, once adapted to a dietary intervention, a single sample may be considered reasonably representative of the microbial community during the time course of a typical trial where the animals are fed a consistent diet.

## Materials and methods

### Sampling

Digesta samples were taken from a selection of 50 experimental animals that were part of two related trials carried out in consecutive years. The trials investigated the long-term effect of different diets and feed additives on CH_4_ emissions, performance and feed efficiency in different breeds of beef cattle during the seven-month finishing phase of production.

The first animal trial, carried out between May 2013 and December 2013 and reported in Troy et al., (2015) [7], involved 32 beef cattle comprising two breeds: crossbred 17 Charolais (CH) and 15 purebred Luing (LU). Over an adaptation period of four weeks, a selection of animals was introduced to the respective diets: 15 to the concentrate-straw based (Concentrate) and 17 to the silage-based (Forage). Individual groups were then allocated to one of three treatments: Control (*n* = 10), Nitrate (*n* = 10), or a high oil (Oil) (*n* = 12) supplement.

The second animal trial, carried out from March 2014 to November 2014 and reported in Duthie et al., (2018) [24] involved 18 beef cattle comprising two breeds: 10 crossbred Aberdeen Angus (AA) and eight crossbred Limousin (LIM). In this case all animals were given a Silage based diet (Forage) and allocated to one of four treatment groups: Control (*n* = 4), Nitrate (*n* = 4), high oil (Oil) (*n* = 4), or combined nitrate and high oil supplements (*n* = 6).

Both studies took place at the Beef and Sheep Research Centre, SRUC, Edinburgh, UK. The experimental work was approved by the Animal Experiment Committee of SRUC and was conducted in accordance with the requirements of the UK Animals (Scientific Procedures) Act 1986. Details on the experimental animals, diet formulation, allocation and sampling time points throughout the course of the experiments including sampling timetables can be found in Table 1 and Additional file 2: Tables S1–2.

At each sampling, approximately 50 mL of rumen liquid were taken by inserting a stomach tube (16 × 2700 mm Equivet Stomach Tube, Jørgen Kruuse A/S, Langeskov, Denmark) nasally and aspirating manually. This liquid was filtered through two layers of muslin and 5 mL strained rumen fluid were mixed with 10 ml phosphate buffered saline containing glycerol (30% v/v). These samples were stored at − 20 °C between collection and analysis.

For short chain fatty acid (SCFA) analysis, a 5 ml sample of the filtered liquid was deproteinised by adding 1 mL metaphosphoric acid (215 g/L) and 0.5 mL methylvaleric acid (10 g/L). Measurements were made using HPLC [48] and expressed as mmol/mol total SCFA.

Methane emissions were measured during the ‘Chamber’ phase (TP5) of the animal trial with animals housed in closed respiration chambers following adaptation in an open training pen. CH_4_ concentrations were measured for each chamber by a multi-gas analyser. CH_4_ production was calculated as the difference between inlet and exhaust gas concentration multiplied by volumetric dry air flow, corrected to standard temperature and pressure (25 °C and 1013 Mbar). Daily CH_4_ production was calculated as the average of individual values and converted to a mass basis. Feed intake was monitored during this phase and methane emissions calculated per day (g/day) and relative to kg dry matter intake (g/kg DMI).

Feed efficiency was calculated using two metrics: Feed conversion ratio (FCR) was calculated as average dry matter intake (DMI) per day (kg/d)/ average daily gain (ADG).

Residual feed intake (RFI) [49] was calculated as deviation of actual DMI (kg/d) from DMI predicted based on linear regression of actual DMI on ADG, mid-metabolic body weight (MBW = BW^0.75^) and FD1 (fat depth at the 12/13th rib at the end of TP4) [50].

### 16S rRNA gene amplicon library preparation

DNA was extracted following the protocol based on Yu and Morrison (2004) [51] by repeated bead-beating followed by precipitation, elution and purification using columns from the QIAamp® DNA Stool Mini Kit, (QIAGEN Ltd., Manchester, UK).

PCR amplification (20 cycles) was carried out in quadruplicate 25 μL reactions using Q5® High-Fidelity DNA polymerase (New England Biolabs Inc.,Hitchin, UK) with universal prokaryotic primers targeting the V4 region of the 16S rRNA gene [52]. Individual samples were identified using unique 12 nucleotide barcodes built into the forward primer. PCR products were cleaned and quantitated using the Qubit high sensitivity dsDNA assay kit (Fisher Scientific UK Ltd., Loughborough, UK). The samples were pooled in equimolar quantities and 80 μL run on a 1% w/v agarose/TBE gel to separate residual primers and dNTPs. The band at the expected size containing the amplicons was cut and purified using a Promega Wizard® SV Gel purification kit (Promega UK, Southampton, UK).

The libraries were quality assessed using an Agilent 2100 Bioanalyzer System (Agilent Technologies. Santa Clara, CA, US) and sequenced by Edinburgh Genomics using Illumina MiSeq v2 250 paired end reagent kits (Illumina UK, Cambridge, UK.). Raw sequence data was uploaded to the European Nucleotide Archive under study accession numbers PRJEB31107 and PRJEB31085.

### Sequence analysis

Sequence data was analysed using mothur 1.39.0 [53] with steps to assemble paired end sequences, remove low quality sequences using both quality control metrics and chimera removal using UCHIME 4.2.40 [54]. Sequence counts in each library were normalised by subsampling to 20,000 sequences per sample.

An operational taxonomic unit (OTU) based approach was selected over phylotyping. This approach better described the microbial community diversity irrespective of whether a taxonomic label could be applied to the representative sequence [55]. This was also important for determination of discriminant taxa where the same phylotype (for example, *Prevotella*) can be associated with opposing treatment or phenotypic groups [56].

Sequences were clustered into OTUs using OptiClust [57] at 97% identity, singletons removed and taxonomic classification of the representative sequences initially using the SILVA 128 SEED reference database [58]. For verification purposes, classifications were subsequently also carried out using the Greengenes (gg_13_8_99) [59] and Ribosomal Database Project (version 16) reference databases [60], and NCBI BLASTn against the complete NCBI reference database. OTUs assigned to the *Archaea* domain were reclassified using the RIM DB taxonomic framework for methanogenic archaea [61].

### Statistical analysis

Sequence counts in each library were normalised by subsampling to 20,000 sequences per sample prior to statistical analysis. Microbial community data was tested for coverage per sample using Good’s statistic [62]. Microbial community species richness and diversity was summarised using the number of observed OTUs and Shannon diversity index (H′), respectively. Beta diversity was calculated using the Bray Curtis dissimilarity metric.

Significance differences of the beta diversity comparisons were given at values of *p* < 0.001. The Parsimony test in mothur was used to assess significance in the dendrogram, and analysis of molecular variance (AMOVA) for the distance matrix used to create the nonlinear multidimensional scaling (NMDS) plot of the total sample data.

Taxonomic biomarkers associated with respective treatment groups were determined using Linear Discriminant Analysis (LEfSe) [63] with a cut off of effect size set at values > 4.0. This was applied to diet, groups, supplement groups and highest and lowest quartile residual feed intake groups.

General statistical analysis was carried out using R version 3.5.1 [25, 64]. Normality of data was determined using quantile-quantile (q-q) plots. The Kruskal-Wallis rank sum test was used to determine significance where data was not normally distributed and Analysis of Variance (ANOVA) was used in instances where data was normally distributed. Repeated measures ANOVA was used for longitudinal data following individual animals over time. The Tukey HSD post hoc test was used to compare multiple treatments. In cases where OTU sequence counts were used for statistical comparison, the data were transformed using square root relative abundance. Correlations were determined using Spearman rank correlation. Significance was given at values of *p* < 0.05 in the case of phenotype data and *p* < 0.001 for sequence data.

## Supplementary information

**Additional file 1.** Table of operational taxonomic units with animal metadata and taxonomic classification.

**Additional file 2.** Supporting figures, tables and statistical analyses not included in the manuscipt.

## Data Availability

Sequence data for the 2013 study samples is available from the European Nucleotide Archive under study accession number PRJEB31107, and the data from the 2014 samples under study accession number PRJEB31085.

## Abbreviations

16S rRNA: 16 Svedberg ribosomal ribonucleic acid
ADG: Average daily gain
AMOVA: Analysis of molecular variance
ANOVA: Analysis of variance
BLAST: Basic local alignment search tool
bp: Base pair
DMI: Dry matter intake
DNA: Deoxyribonucleic acid
dNTP: Deoxynucleotide Triphosphate
FCR: Feed conversion ratio
LDA: Linear discriminant analysis
LEfSe: Linear discriminant analysis effect size
NCBI: National center for biotechnology Information
NMDS: Nonlinear multidimensional scaling
nt: Nucleotide
OTU: Operational taxonomic unit
PCR: Polymerase chain reaction
RDP: Ribosomal database project
RFI: Residual feed intake
RIM DB: Rumen and intestinal methanogen-data base
SCFA: Short chain fatty acid
TBE: Tris borate ethylenediaminetetraacetic acid
TP: Time point
