# Supplementary material for: Temporal stability of the rumen microbiota in beef cattle, and response to diet and supplements
Source: Anim Microbiome. 2019 Nov 19;1:16. doi: 10.1186/s42523-019-0018-y (PMC7807515; doi:10.1186/s42523-019-0018-y)

**Additional File 2**

**Table S1.** Experimental Animals Diets, Supplements and Phenotype Data

| Animal | UK ID | Breed | Year | Diet | Supplement | FCR | RFI | CH_4_ g/Day | CH_4_ g/kg DMI |
| --- | --- | --- | --- | --- | --- | --- | --- | --- | --- |
|  |  |  |  |  |  |  |  |  |  |
| 1 | 202309 | Luing | 2013 | Forage | Nitrate | 8.6667914 | 0.7999285 | 158.2678164 | 17.9645649 |
| 2 | 602348 | Luing | 2013 | Forage | Nitrate | 7.1088144 | 0.2675523 | 209.9152981 | 18.1587628 |
| 3 | 600985 | CHx | 2013 | Forage | Nitrate | 8.6968865 | 1.0097021 | 206.4445661 | 25.96787 |
| 4 | 701235 | CHx | 2013 | Forage | Nitrate | 5.9957988 | -0.173446 | 235.7226554 | 22.9302194 |
| 5 | 101053 | Luing | 2013 | Concentrate | Nitrate | 8.5682363 | -0.3318918 | 123.2876667 | 18.8513252 |
| 6 | 201009 | CHx | 2013 | Concentrate | Nitrate | 9.1168176 | 0.43714 | 153.8431764 | 20.9310444 |
| 7 | 500677 | CHx | 2013 | Concentrate | Nitrate | 5.8480091 | -1.1720002 | 79.3594743 | 12.6975159 |
| 8 | 702314 | Luing | 2013 | Concentrate | Nitrate | 6.9663827 | -1.5171322 | 107.1045904 | 16.6053629 |
| 9 | 202281 | Luing | 2013 | Forage | Control | 9.645938 | 0.4124067 | 238.6185323 | 29.6420537 |
| 10 | 301548 | CHx | 2013 | Forage | Control | 9.215746 | -0.5274059 | 250.865476 | 27.120592 |
| 11 | 301017 | CHx | 2013 | Concentrate | Control | 7.0014493 | -0.3959296 | 175.3606325 | 15.2753164 |
| 12 | 401549 | CHx | 2013 | Concentrate | Control | 8.5666309 | 0.5195846 | 170.3095294 | 16.0065347 |
| 13 | 502291 | Luing | 2013 | Concentrate | Control | 11.5379703 | 0.4706358 | 207.9608654 | 18.162521 |
| 14 | 402283 | Luing | 2013 | Concentrate | Control | 8.0303406 | 0.1877723 | 235.0301051 | 19.3599757 |
| 15 | 502256 | Luing | 2013 | Forage | Control | 7.3125478 | 0.1160638 | 266.4295176 | 28.9912424 |
| 16 | 701007 | CHx | 2013 | Forage | Control | 6.2279959 | -0.1159922 | 248.3558562 | 24.2535016 |
| 17 | 202434 | Luing | 2013 | Concentrate | Oil | 11.9113142 | -0.4577346 | 149.8263116 | 12.7079145 |
| 18 | 401011 | CHx | 2013 | Concentrate | Oil | 7.0480792 | 0.2149627 | 134.7089933 | 13.0027986 |
| 19 | 601544 | CHx | 2013 | Concentrate | Oil | 10.2883027 | -0.4229429 | 172.8767929 | 14.6879178 |
| 20 | 702293 | Luing | 2013 | Concentrate | Oil | 8.0960157 | -0.8927011 | 138.1050593 | 17.1986375 |
| 21 | 500991 | CHx | 2013 | Forage | Oil | 6.4666595 | 0.2814275 | 191.7580526 | 21.915206 |
| 22 | 701200 | CHx | 2013 | Forage | Oil | 7.1534264 | -0.3466332 | 244.5086015 | 22.535355 |
| 23 | 401084 | Luing | 2013 | Forage | Oil | 7.3557783 | 0.4357097 | 248.1832519 | 21.7895744 |
| 24 | 701059 | Luing | 2013 | Forage | Oil | 9.0915361 | 0.2742009 | 204.7615382 | 18.5977782 |
| 25 | 301024 | CHx | 2013 | Forage | Oil | 7.0655249 |  |  | 22.7875759 |
| 26 | 401232 | CHx | 2013 | Forage | Oil | 6.530965 |  |  |  |
| 27 | 600678 | CHx | 2013 | Forage | Oil | 6.7025301 |  |  | 25.2744503 |
| 28 | 501071 | Luing | 2013 | Concentrate | Nitrate | 7.0044354 |  |  | 10.8045115 |
| 29 | 602396 | Luing | 2013 | Forage | Nitrate | 9.1524984 |  |  | 20.6956964 |
| 30 | 300689 | CHx | 2013 | Concentrate | Oil | 7.5936458 |  |  | 15.8880723 |
| 31 | 602327 | Luing | 2013 | Concentrate | Control | 10.0201144 |  |  | 20.3223203 |
| 32 | 601227 | CHx | 2013 | Forage | Control | 6.6559144 |  |  | 33.5873141 |
|  |  |  |  |  |  |  |  |  |  |
| 1 | 201490 | LIM | 2014 | Forage | Control | 5.1554398 | -1.2797796 | 257.0081186 | 28.5305938 |
| 2 | 301519 | LIM | 2014 | Forage | Oil | 5.5033709 | -0.8238953 | 190.8222261 | 26.5942565 |
| 3 | 702677 | AA | 2014 | Forage | Combined | 6.0861323 | -0.8974657 | 222.4555887 | 23.2826285 |
| 4 | 202672 | AA | 2014 | Forage | Oil | 6.2240349 | 0.2943832 | 237.5198471 | 22.1730638 |
| 5 | 202581 | AA | 2014 | Forage | Nitrate | 6.3888036 | -0.1590707 | 230.2502327 | 24.8081893 |
| 6 | 502626 | LIM | 2014 | Forage | Nitrate | 6.4177986 | -1.7554339 | 246.108618 | 22.9071652 |
| 7 | 602732 | AA | 2014 | Forage | Combined | 6.4490353 | 0.0663335 | 200.1655312 | 17.5078735 |
| 8 | 502640 | AA | 2014 | Forage | Control | 6.5357582 | 0.0195113 | 222.4661967 | 21.3318489 |
| 9 | 702733 | AA | 2014 | Forage | Combined | 7.288278 | 0.335928 | 240.3089066 | 21.9435675 |
| 10 | 502605 | AA | 2014 | Forage | Control | 7.7075072 | 1.4447628 | 279.3474503 | 22.3635544 |
| 11 | 602662 | LIM | 2014 | Forage | Control | 7.8542102 | 0.2949739 | 233.7637074 | 22.1006444 |
| 12 | 305449 | AA | 2014 | Forage | Oil | 8.3079019 | 1.0001694 | 278.945617 | 26.4206291 |
| 13 | 202665 | LIM | 2014 | Forage | Combined | 8.7954731 | 0.5758924 | 200.3496579 | 21.6359725 |
| 14 | 705460 | AA | 2014 | Forage | Nitrate | 8.8528529 | 4.1623815 | 292.6787143 |  |
| 15 | 102692 | LIM | 2014 | Forage | Nitrate | 8.8564547 | 0.158189 | 208.3684442 | 18.652236 |
| 16 | 105454 | AA | 2014 | Forage | Combined | 6.8608272 | 0.4037656 | 234.8741898 | 18.0379965 |
| 17 | 401471 | LIM | 2014 | Forage | Combined | 8.4803566 | -0.6876687 | 173.6507779 | 25.0275539 |
| 18 | 502661 | LIM | 2014 | Forage | Oil | 7.6952825 | -0.053287 | 245.8700657 | 24.5308849 |

**Table S2.** Ingredient composition of mixed forage: concentrate (Forage) and high-concentrate (Concentrate) diets (g/kg DM).

|  | **Nutribeef 2013 Forage** | | | **NutriBeef 2013 Concentrate** | | |
| --- | --- | --- | --- | --- | --- | --- |
| **Ingredient** | **Control** | **Nitrate** | **Lipid** | **Control** | **Nitrate** | **Lipid** |
| **Silage** | 189 | 193 | 192 | 0 | 0 | 0 |
| **WCBS** | 312 | 316 | 315 | 0 | 0 | 0 |
| **Barley Straw** | 0 | 0 | 0 | 84 | 82 | 80 |
| **Bruised Barley** | 340 | 392 | 296 | 739 | 803 | 700 |
| **RSM** | 128 | 43 | 7 | 146 | 57 | 10 |
| **Calcinit** | 0 | 27 | 0 | 0 | 26 | 0 |
| **RSC (Lipid)** | 0 | 0 | 160 | 0 | 0 | 179 |
| **Molasses** | 20 | 21 | 20 | 21 | 21 | 21 |
| **Minerals*** | 10 | 9 | 10 | 10 | 10 | 10 |

|  | **NutriBeef 2014 Forage** | | | |
| --- | --- | --- | --- | --- |
| **Ingredient** | **Control** | **Nitrate** | **Lipid** | **Combined** |
| **Silage** | 210 | 211 | 209 | 210 |
| **WCBS** | 347 | 347 | 346 | 346 |
| **Barley Straw** | 0 | 0 | 0 | 0 |
| **Bruised Barley** | 336 | 388 | 289 | 263 |
| **RSM** | 79 | 0 | 0 | 0 |
| **Calcinit** | 0 | 25 | 0 | 25 |
| **MDG (Lipid)** | 0 | 0 | 128 | 127 |
| **Molasses** | 19 | 20 | 19 | 19 |
| **Minerals*** | 9 | 9 | 9 | 9 |

Silage, grass silage; WCBS, whole crop barley silage; Barley, barley grain; RSM, rapeseed meal; MDG, maize dark grains; Calcinit, calcium nitrate; RSC rapeseed cake.

*Contained (mg/kg): Fe, 6036; Mn, 2200; Zn, 2600; Iodine, 200; Co, 90; Cu, 2500; Se 30; (μg/kg): vitamin E, 2000; vitamin B12, 1000; vitamin A, 151515; vitamin D, 2500

**Table S3**. Comparison of microbial communities between basal diet and treatment groups using analysis of molecular variance (AMOVA).

|  | **F Value** | **P - Value Summary** |
| --- | --- | --- |
| Concentrate Control - Concentrate Nitrate | 4.78524 | p - value: <0.001* |
| Concentrate Control - Concentrate Oil | 2.28754 | p - value: 0.027 |
| Concentrate Control - Concentrate PreTreatment | 2.21753 | p - value: 0.017 |
| Concentrate Control - Forage Combined | 16.7023 | p - value: <0.001* |
| Concentrate Control - Forage Control | 34.8092 | p - value: 0.001* |
| Concentrate Control - Forage Nitrate | 37.8226 | p - value: 0.001* |
| Concentrate Control - Forage Oil | 23.6384 | p - value: <0.001* |
| Concentrate Control - Forage PreTreatment | 15.371 | p - value: <0.001* |
| Concentrate Nitrate - Concentrate Oil | 5.63874 | p - value: <0.001* |
| Concentrate Nitrate - Concentrate PreTreatment | 5.9474 | p - value: <0.001* |
| Concentrate Nitrate - Forage Combined | 17.3924 | p - value: <0.001* |
| Concentrate Nitrate - Forage Control | 33.4985 | p - value: <0.001* |
| Concentrate Nitrate - Forage Nitrate | 36.5591 | p - value: <0.001* |
| Concentrate Nitrate - Forage Oil | 22.2495 | p - value: <0.001* |
| Concentrate Nitrate - Forage PreTreatment | 15.8886 | p - value: <0.001* |
| Concentrate Oil - Concentrate PreTreatment | 4.56581 | p - value: <0.001* |
| Concentrate Oil - Forage Combined | 23.9963 | p - value: <0.001* |
| Concentrate Oil - Forage Control | 47.1134 | p - value: <0.001* |
| Concentrate Oil - Forage Nitrate | 52.1654 | p - value: <0.001* |
| Concentrate Oil - Forage Oil | 32.7303 | p - value: <0.001* |
| Concentrate Oil - Forage PreTreatment | 22.2639 | p - value: <0.001* |
| Concentrate PreTreatment - Forage Combined | 8.37168 | p - value: <0.001* |
| Concentrate PreTreatment - Forage Control | 13.6701 | p - value: <0.001* |
| Concentrate PreTreatment - Forage Nitrate | 15.913 | p - value: <0.001* |
| Concentrate PreTreatment - Forage Oil | 10.7013 | p - value: <0.001* |
| Concentrate PreTreatment - Forage PreTreatment | 7.42347 | p - value: <0.001* |
| Forage Combined - Forage Control | 3.93656 | p - value: <0.001* |
| Forage Combined - Forage Nitrate | 6.76732 | p - value: <0.001* |
| Forage Combined - Forage Oil | 2.0014 | p - value: 0.031 |
| Forage Combined - Forage PreTreatment | 1.72642 | p - value: 0.048 |
| Forage Control - Forage Nitrate | 3.38194 | p - value: <0.001* |
| Forage Control - Forage Oil | 2.30194 | p - value: 0.008 |
| Forage Control - Forage PreTreatment | 2.30948 | p - value: 0.002 |
| Forage Nitrate - Forage Oil | 4.33501 | p - value: <0.001* |
| Forage Nitrate - Forage PreTreatment | 3.86098 | p - value: <0.001* |
| Forage Oil - Forage PreTreatment | 1.5689 | p - value: 0.074 |

**Figure S1**. Relative abundance of microbial families associated with Concentrate and Forage basal diets. Note that the dataset contains sequences that may not have been classifiable down to the Family-level, but which could be confidently classified and grouped together at higher taxonomic levels.

A)

**
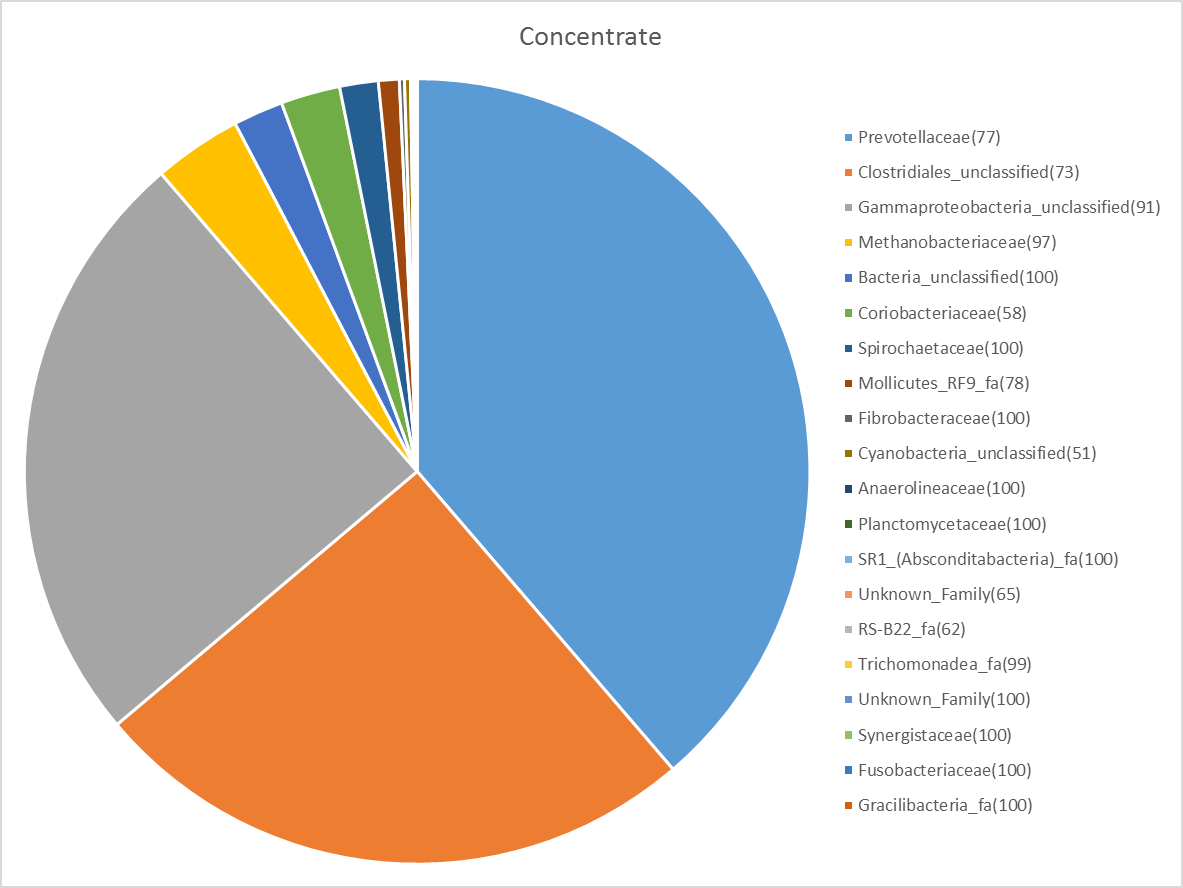
**

B)


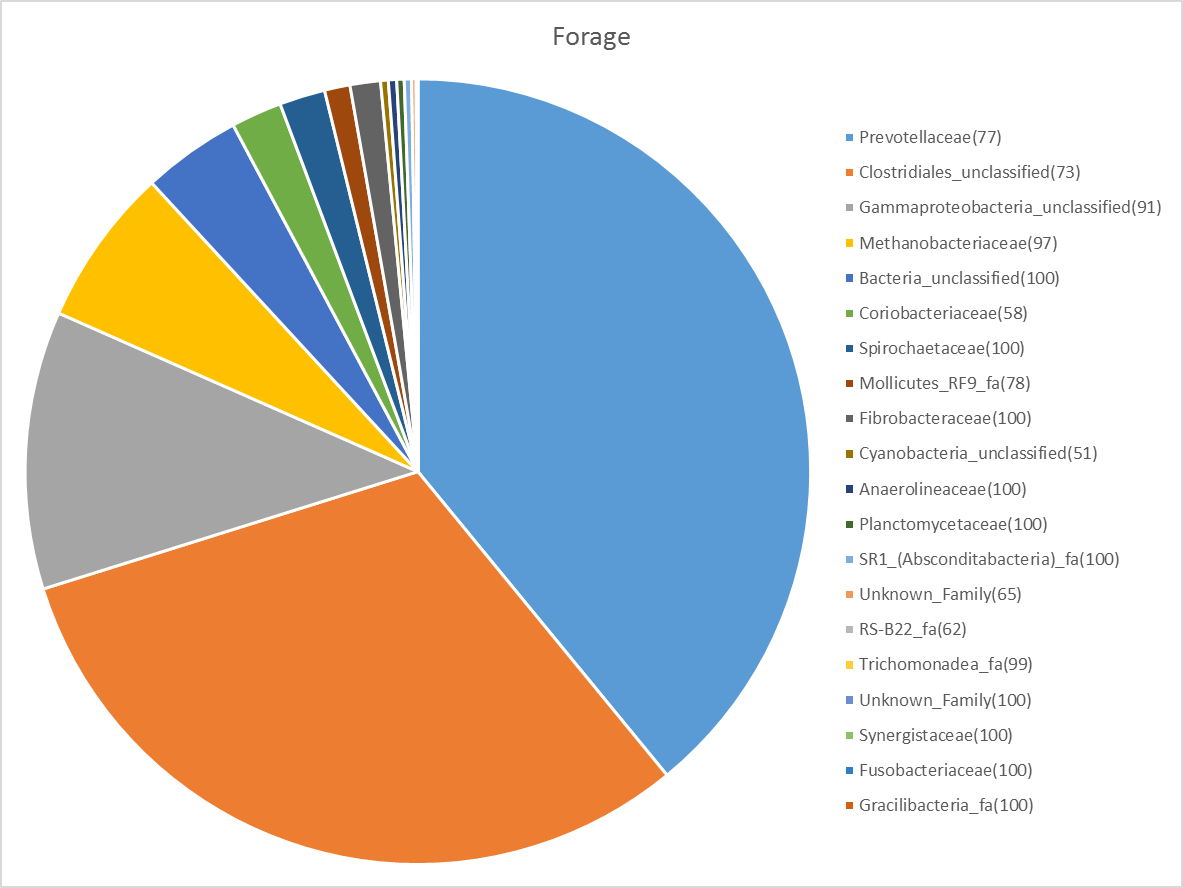


**Figure S2**. Comparison of microbial diversity between Forage (Blue) and Concentrate (Orange) fed cattle (Shannon Index significantly lower in concetrate fed animals (p<0.001)).


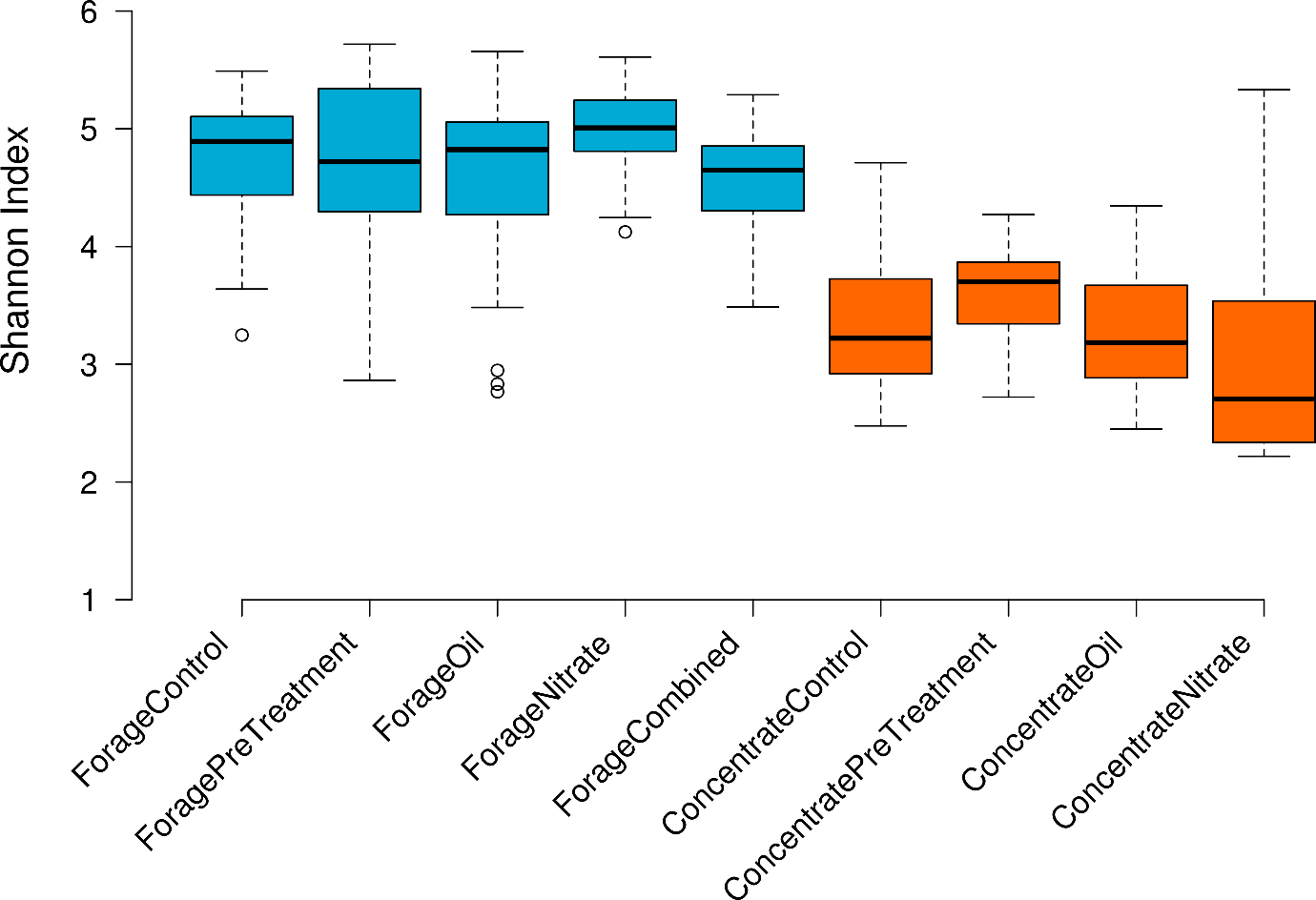


**Figure S3**. Stability of the microbial communty. Microbial diversity (Shannon Index) measured during the 200 day finishing period. Orange: Cattle fed high concetrate basal diet. Blue: Cattle fed forage basal diet.


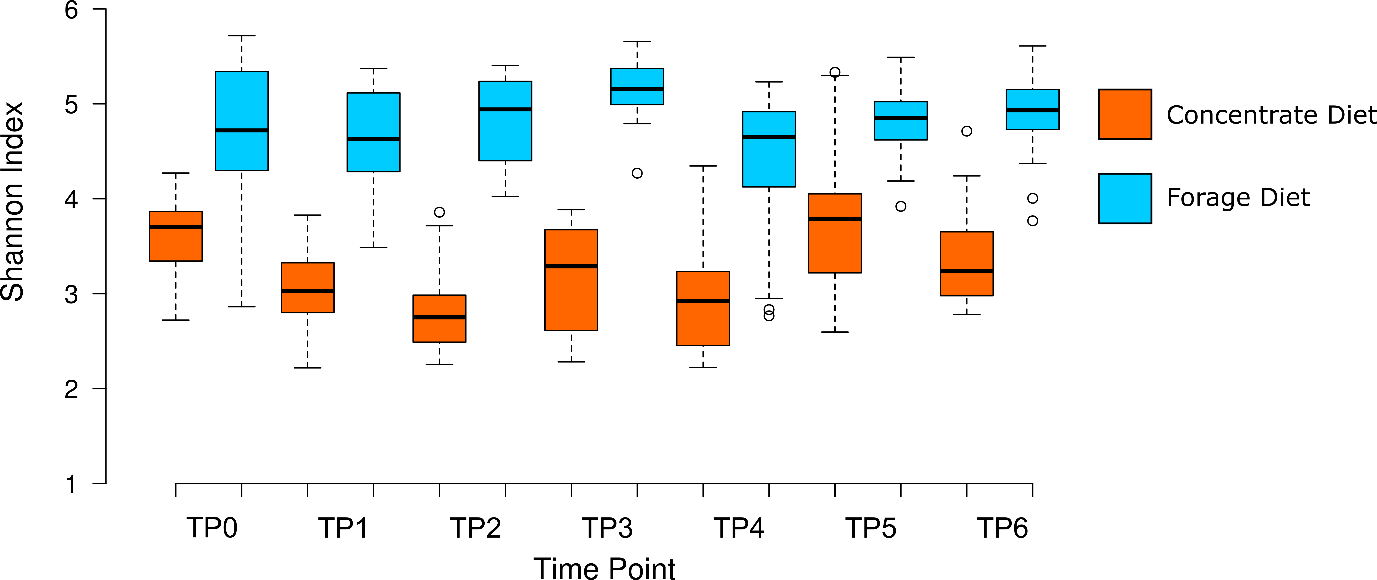


**Figure S4**. Comparison of microbial diversity (Shannon Index) between Forage 2013 and Forage 2014 animal trials. Average microbial diversity was significantly higher (p<0.001) in forage fed cattle during the 2013 animal trial.


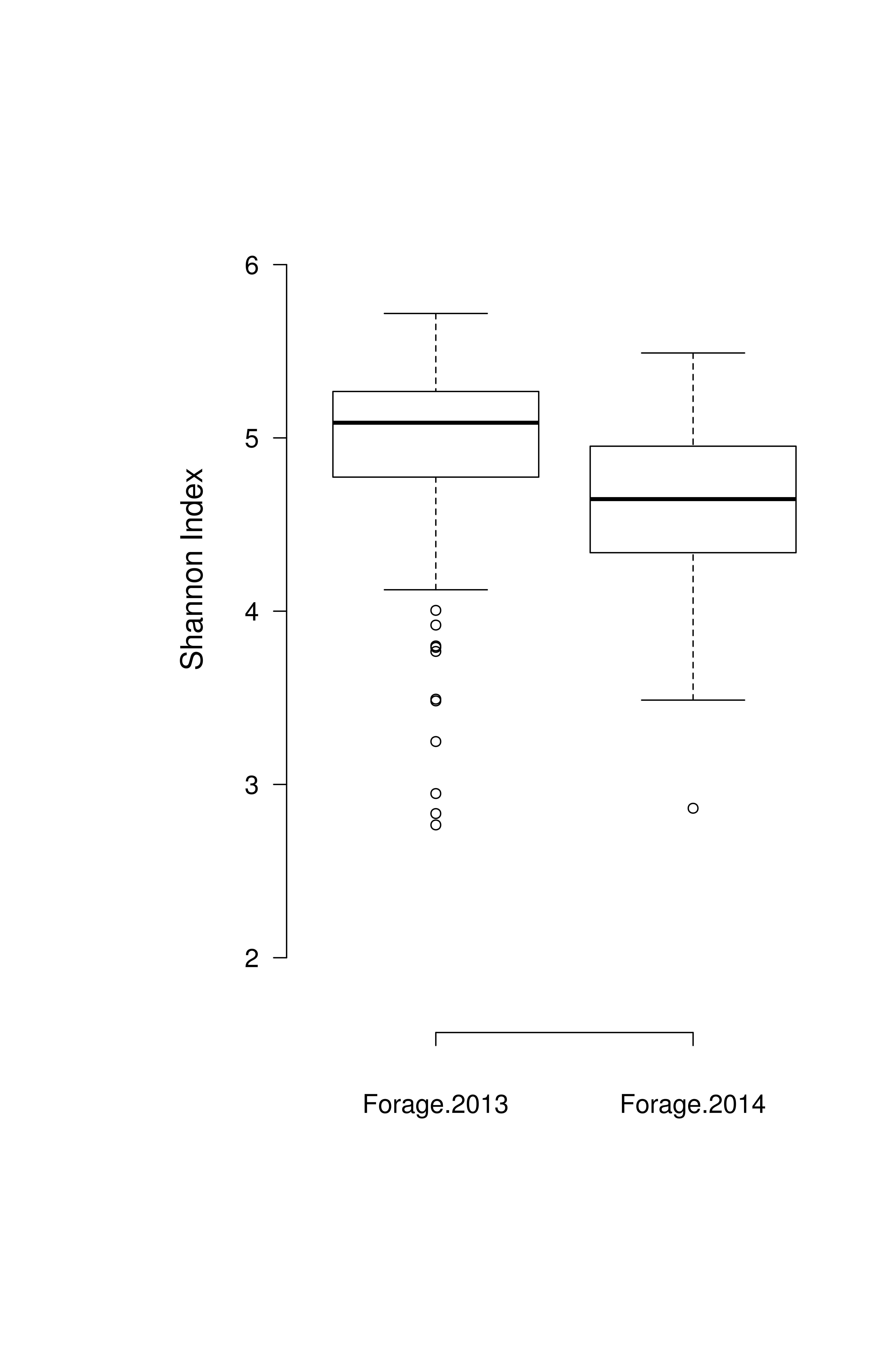


**Figure S5**. Neighbour Joining (Saitou and Nei 1987) phylogenetic analysis of OTUs assigned to *Proteobacteria* biomarkers with associations with either concentrate or forage basal diets (LDA Effect size >2.0). Tree constructed using MEGA5. Pairwise alignment of sequences carried out using MUSCLE, consensus tree generated from 1000 bootstrap iterations and rooted with *E. coli* as an outgroup.

Abundance of OTU relative to the total microbial community. Balance of OTU in animals fed ● Concentrate or ● Forage basal diets.


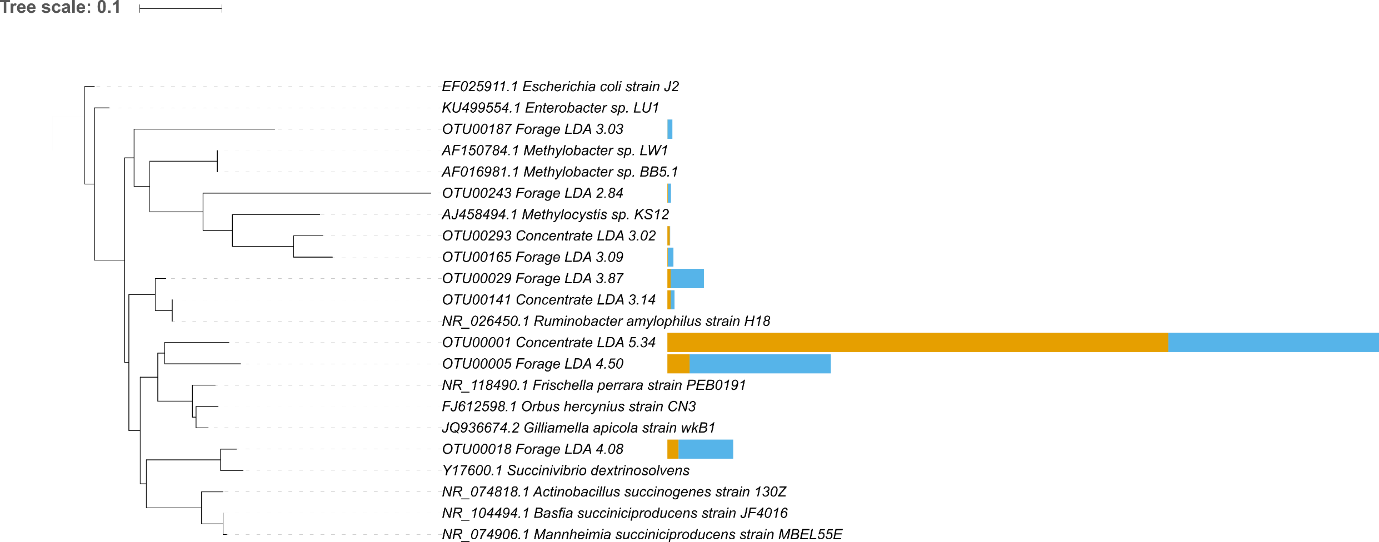


**Figure S6**. Scatter Matrix: Pairwise comparison of Bray Curtis dissimilarity between time points. All pairwise comparisons of beta diversity between time points significantly correlated (Spearman Rank p<0.001).

Time Points: TP0 – Pre-treatment, TP1 Adaptation, TP2 – Performance Test Start, TP3 – Performance Test Mid, TP4 – Performance Test End, TP5 – Methane Chamber, TP6 – Slaughter.


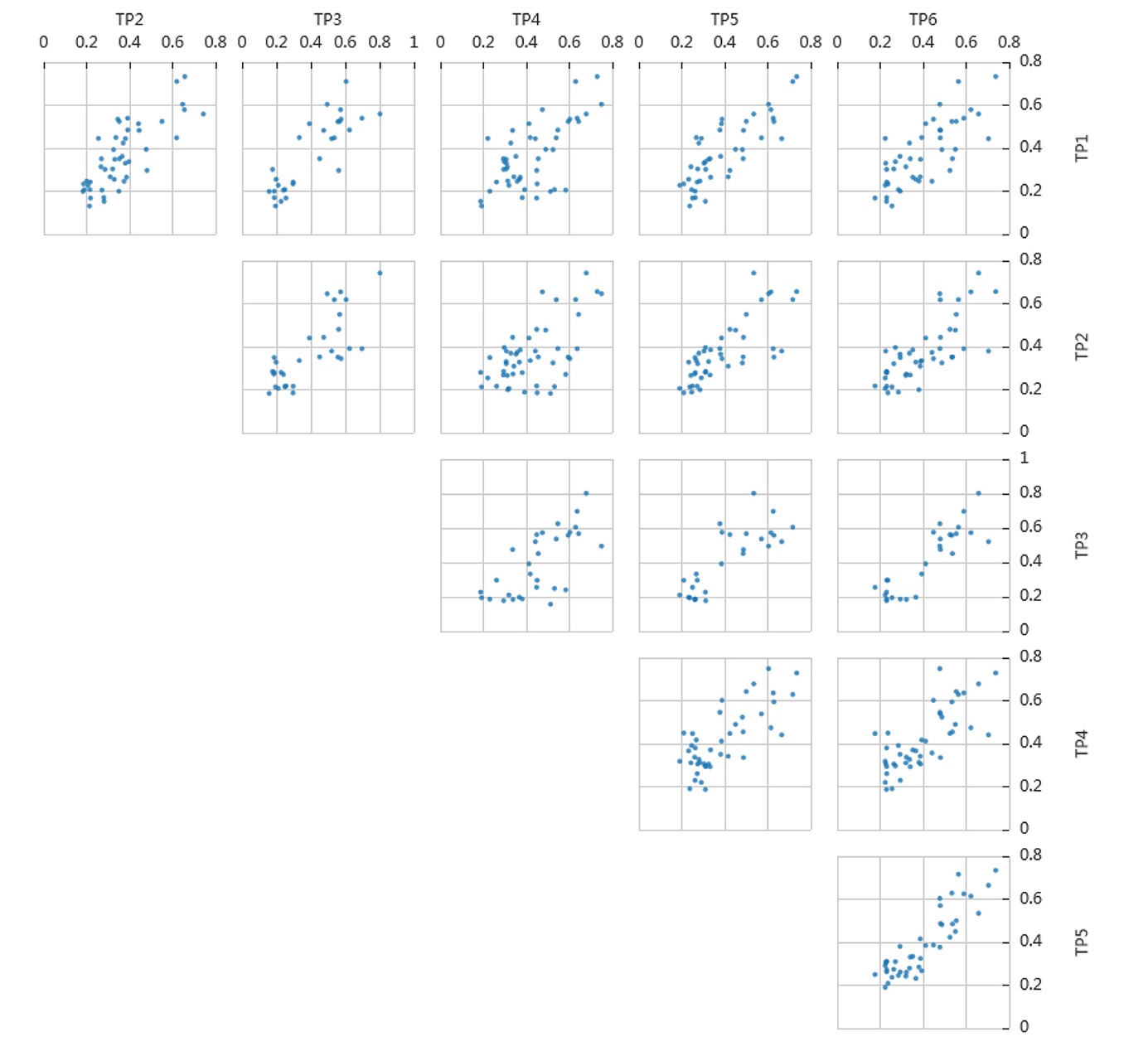

Supplement: Supplementary file 2 — Additional file 2. Supporting figures, tables and statistical analyses not included in the manuscipt. [file 42523_2019_18_MOESM2_ESM.docx]
